# Sleep Duration and Physical Activity Profiles Associated With Self-Reported Stroke in the United States: Application of Bayesian Belief Network Modeling Techniques

**DOI:** 10.3389/fneur.2018.00534

**Published:** 2018-07-19

**Authors:** Azizi A. Seixas, Dwayne A. Henclewood, Stephen K. Williams, Ram Jagannathan, Alberto Ramos, Ferdinand Zizi, Girardin Jean-Louis

**Affiliations:** ^1^Department of Population Health, Department of Psychiatry, NYU Langone Health, New York, NY, United States; ^2^Booz Allen Hamilton, Boston, MA, United States; ^3^Emory University Rollins School of Public Health, Atlanta, GA, United States; ^4^Department of Neurology, Miller School of Medicine, University of Miami, Miami, FL, United States

**Keywords:** sleep duration, physical activity, stroke, machine learning, sex

## Abstract

**Introduction:** Physical activity (PA) and sleep are associated with cerebrovascular disease and events like stroke. Though the interrelationships between PA, sleep, and other stroke risk factors have been studied, we are unclear about the associations of different types, frequency and duration of PA, sleep behavioral patterns (short, average and long sleep durations), within the context of stroke-related clinical, behavioral, and socio-demographic risk factors. The current study utilized Bayesian Belief Network analysis (BBN), a type of machine learning analysis, to develop profiles of physical activity (duration, intensity, and frequency) and sleep duration associated with or no history of stroke, given the influence of multiple stroke predictors and correlates. Such a model allowed us to develop a predictive classification model of stroke which can be used in post-stroke risk stratification and developing targeted stroke rehabilitation care based on an individual's profile.

**Method:** Analysis was based on the 2004–2013 National Health Interview Survey (*n* = 288,888). Bayesian BBN was used to model the omnidirectional relationships of sleep duration and physical activity to history of stroke. Demographic, behavioral, health/medical, and psychosocial factors were considered as well as sleep duration [defined as short < 7 h. and long ≥ 9 h, referenced to healthy sleep (7–8 h)], and intensity (moderate and vigorous) and frequency (times/week) of physical activity.

**Results:** Of the sample, 48.1% were ≤ 45 years; 55.7% female; 77.4% were White; 15.9%, Black/African American; and 45.3% reported an annual income < $35 K. Overall, the model had a precision index of 95.84%. We found that adults who reported 31–60 min of vigorous physical activity six times for the week and average sleep duration (7–8 h) had the lowest stroke prevalence. Of the 36 sleep (short, average, and long sleep) and physical activity profiles we tested, 30 profiles had a self-reported stroke prevalence lower than the US national average of approximately 3.07%. Women, compared to men with the same sleep and physical activity profile, appeared to have higher self-reported stroke prevalence. We also report age differences across three groups 18–45, 46–65, and 66+.

**Conclusion:** Our findings indicate that several profiles of sleep duration and physical activity are associated with low prevalence of self-reported stroke and that there may be sex differences. Overall, our findings indicate that more than 10 min of moderate or vigorous physical activity, about 5–6 times per week and 7–8 h of sleep is associated with lower self-reported stroke prevalence. Results from the current study could lead to more tailored and personalized behavioral secondary stroke prevention strategies.

## Introduction

Several meta-analytical studies indicate that physically active individuals are less likely to have a stroke compared with their sedentary counterparts (1–3). Physical activity attenuates stroke risk by improving sub-clinical risk factors such as endothelial function, high cholesterol, elevated body mass index, blood clots, and restricted blood flow (4–10). Additionally, there is growing evidence that sleep duration, such as short (short ≤ 6 h) or long (≥9 h) sleep durations, insomnia, or obstructive sleep apnea increase the risk of stroke directly and indirectly (via mediated pathways such as arrhythmias, coronary artery disease, high blood pressure, elevated glucose, cholesterol) (11–13). Despite overwhelming evidence that physical activity and sleep are associated with lower likelihood of stroke, less is known about differential (single) and combined associations of physical activity (intensity, duration, and frequency), sleep, and other health-related lifestyle behaviors on stroke.

Despite compelling evidence that physical inactivity and poor/unhealthy sleep (short and long sleep) are separately associated with stroke (which is the type of knowledge traditional analytical methods provide), less is known about: (1) how physical activity and sleep are proximally, distally, and quadratically associated with stroke and stroke related risk factors, questions that go beyond the constrains of traditional correlation tests and linear relationships between two variables and is unable to capture multiple non-linear relationships between physical activity, sleep, stroke, and stroke risk factors; (2) how stroke related factors mediate or moderate associations among physical activity, sleep and stroke through mutual and joint associations; and (3) what combination of physical activity (intensity, duration, and frequency) and sleep duration (short, average, and long) and stroke related factors are associated and predictive of self-reported stroke prevalence.

Answering such questions require mathematically quantifying the single, combined, and the omni-directional associations among physical activity, sleep duration, stroke, and other stroke-related socio-demographic, behavioral, and chronic disease factors. Performing such analyses go beyond traditional regression models which are limited in capturing omnidirectional and non-linear relationships. Therefore, in the current study, we utilized Bayesian Belief Network (BBN) modeling to accurately capture omnidirectional and non-linear associations among physical activity (intensity, frequency, and duration of activity), sleep, and other stroke-related factors on self-reported stroke. Specifically, we tested which combinations of physical activity, sleep, sex, and age are associated with a stroke prevalence below the United States' national average.

## Materials and methods

### Sample

Analysis was based on data from the 2004 to 2013 National Health Interview Survey (NHIS) dataset. The NHIS dataset is a nationally representative population-based study of non-institutionalized adults from 50 U.S. states. Socio-demographic, behavioral, health conditions and physician-diagnosed chronic disease data were obtained through face-to-face interviews using computer-assisted personal interviewing (CAPI) The final sample consisted of 288,888 individuals (14). This study was carried out in accordance with the recommendations of the Center for Disease Control and Prevention (CDC) data use guidelines of the Ethics Review Board and the National Center for Health Statistics. The protocol was approved by the CDC. All subjects gave written informed consent in accordance with the Declaration of Helsinki. We accessed the de-identified data from the IPUMS Health Survey website at nhis.ipums.org/nhis. Since the data were publicly available and de-identified, no approval was needed from the CDC or NYU's institutional ethics review committee to access and analyze the data for research purposes. We adhered strictly to the data use guidelines of the Ethics Review Board of the CDC and the National Center for Health Statistics. For the reasons cited above, no additional informed consent was needed for the current study.

### Variables

Variables of interest were derived from a two-step process. First, we reviewed the literature on potential stroke correlates and then compiled a list based on whether data were available for the years 2004–2013 in the National Health Interview Survey (NHIS). The list winnowed to 34 variables, which included: (1) target variables—a moderate and vigorous physical activity (e.g., leisurely walking/bicycling, slow swimming/dancing, running, lifting weights, and simple gardening activities), and (b) sleep duration (short, average, and long); and (2) covariates: socio-demographic, general health, chronic health conditions, and health risk behaviors and history of stroke event (see Table 1). We acknowledge that our list of stroke correlates is not exhaustive, since the NHIS has a limited selection of self-reported variables.

**Table 1:** Definition of variables.

**Variable**	**Definitions**
HEALTH	Self-reported health status
AGE	Age reported in years
EMPSTATWKYR	Employed over the past year
HYPERTENEV	Ever been told had hypertension by physician
CHEARTDIEV	Coronary Heart Disease
HEARTATTEV	Heart attack
MARSTAT	Marital status
HEARTCONEV	Congenital heart disease
DIABETICEV	Ever been told had Diabetes by physician
**VIG10DMIN**	Duration of vigorous physical activity (min)
ANGIPECEV	Ever been told had Angina by physician
ALCSTAT1	Alcoholic drinks in a year
ALCSTAT2	Alcoholic drinks in a year
INCFAM97ON2	Annual family income
KIDNEYWKYR	Physician-diagnosed kidney disease
**HRSLEEP**	Average total sleep time in hours
EMPHYSEMEV	Ever been told had emphysema by physician
EDUCREC1	Highest Grade Level Achieved
**MOD10DMIN**	Duration of moderate physical activity (min)
USUALPL	Usual place for medical treatment
EMODISTRESS	Emotional distress
WORKEV	Has person ever worked ever
POORYN	Above or below poverty threshold of the United States
SMOKEV	Ever smoked 100+ cigarettes ever
**MOD10FWK**	Frequency of moderate physical activity (day)
LIVERCONYR	Physician-diagnosed liver condition
RACEA	Self-identified race
HISPETH	Hispanic/Latina ethnicity
**VIG10FWK**	Frequency of vigorous physical activity (days)
HEIGHT	Height in meters
BMICALC	Body mass Index
REGION	Region in the United States
WEIGHT	Weight in pounds
SEX	Male or female

*Bolded Variables are variables of interest for current study*.

#### Self-reported stroke

Individuals were asked whether they ever had a physician diagnosed stroke. Hereon forward we will refer to this variable as “self-reported stroke.”

#### Moderate physical activity

These are activities that “cause only light sweating or a slight to moderate increase in breathing or heart rate.” Examples of moderate physical activity include leisure walking or bicycling, slow swimming or dancing, and light gardening. Participants were also asked about the duration and frequency of their activity.

#### Vigorous physical activity

These are activities that “cause heavy sweating or large increases in breathing or heart rate.” Examples of vigorous physical activity include fast walking, fast bicycling, jogging, strenuous swimming or sports play, vigorous aerobic dance, and strenuous gardening. Participants were also asked about the duration and frequency of their activity.

#### Sleep duration

Participants were asked “On average, how many hours of sleep do you get in a 24-h period.” Hours of sleep were entered in whole numbers. Sleep duration reports greater than or equal to 30 min (half hour) were rounded up to the next whole number and less than or equal to 29 min were dropped. For example, total sleep time of 6 h. and 45 min was rounded up to a total sleep time of 7 h, and a total sleep time of 6 h and 5 min was rounded down to a total sleep time of 6 h.

### Research question

The aim of the study was to test and experiment *in silico*, through machine learning and dynamic observational inference from the machine-learnt model, the following research questions: (1) how are physical activity and sleep proximally, distally, and quadratically associated with self-reported stroke and stroke correlates; (2) how stroke-related correlates mediate or moderate the associations of physical activity, sleep and self-reported stroke through mutual and joint associations; and (3) what combination of physical activity (intensity, duration, and frequency), sleep duration (short, average and long) and stroke-related factors are associated with the lowest prevalence of self-reported stroke.

### Building model through machine learning

To test the above questions, we utilized Bayesian Belief Network (BBN) machine learning to develop a model of 34 stroke correlates with self-reported stroke (Table 1). Unlike traditional regression analysis models, BBN allows us to accurately capture multiple omnidirectional relationships in a highly dimensional model. BBNs has the potential to revolutionize how we conceptualize inference, specifically causal inference, as it provides an efficient method of assessing proximal, distal, direct, indirect, and omnidirectional relationships in one model. BBN models are represented by directed acyclic graphs (DAGs), which are computationally learned networks of nodes (variables). DAGs represent the mutual dependence and the joint probability distribution (JPD) of two or more variables. Mutual dependence measures how much of variable A explains variable B and vice versa and probability estimates determine mutual dependencies throughout the BBN model. Through BBN models, the analyst[s] is able to observe the impact of a given variable[s], such as discrete categorical bins, on the entire network (joint distribution of model) or a particular variable. Though, BBN models allow us to ask unique questions and test omnidirectional relationships, there are some limitations that we acknowledge in the limitation section below.

We used the BayesiaLab statistical software package version 6.0.8 to develop the BBN model (14, 15) and performs all analyses. BayesiaLab offers a suite of unsupervised and supervised machine learning algorithms that may be employed to derive the conditional probabilities of highly dimensional problem. The software also allowed us to rigorously impute missing data, which was pivotal since missing values are common in large population-based data sets like the NHIS (14, 15). In brief, imputed missing values were derived from: (1) the machine learnt BBN model and (2) whether the participant's characteristics are closely aligned with other participants in the sample with similar variables characteristics.

## Analytical workflow

For the current study, we performed a series of data preparation, model validation and calibration, exploratory correlation, supervised and unsupervised learning and observational inference methods to test our research questions.

### Preparing the data and model validation

**Figure 1:**
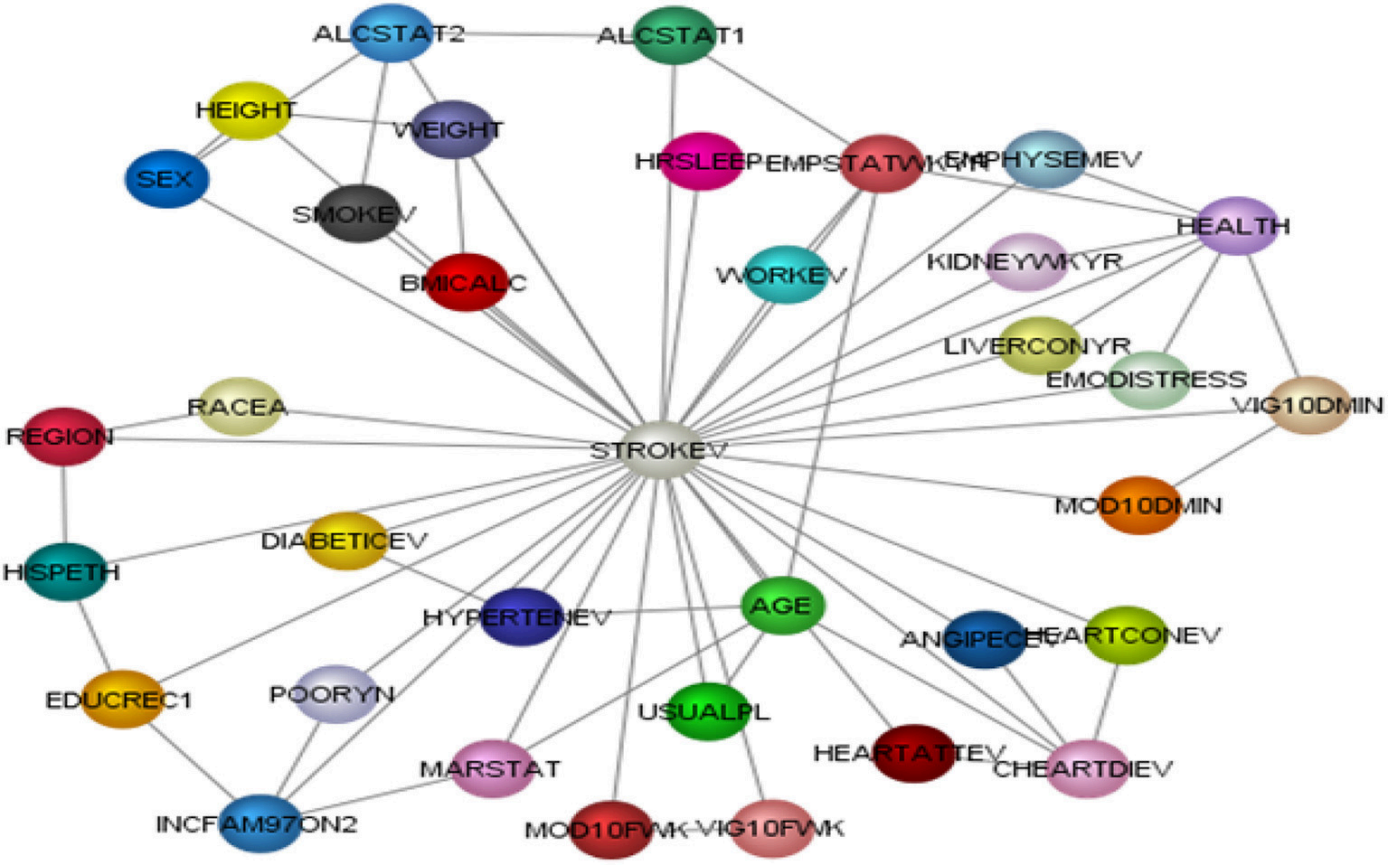
Model structure of Bayesian belief networkof stroke event.

Two of the core phases of preparing data include: (1) pre-processing the data which entails downloading data with CDC and NHIS sample weights, defining categorical and continuous variables, and assessing for valid and missing data entries; and (2) binning the variables in their most appropriate bins. We broke the data into two models, a learning/training model and test model. Cases are randomly selected to the two models by the software's (BayesiaLab) random selection process. The learning/training model which consisted of 231,111 cases was used to learn omni-directional relationships among variables using supervised learning, while test model which consisted of 57,777 was used to evaluate and validate parameters and structure of the BBN learning/training model. For the current model, we assessed six different algorithms and determined that the Augmented Naïve Bayes algorithm provided the best minimum description length, a marker of fit and complexity of model (Figure 1).

Afterwards, we assessed whether our BBN model accurately and precisely determined relationships amongst self-reported stroke (target variable) and 34 other variables. The test model had an overall precision of 96.24% and an overall reliability of 95.46%. Our learning/training model had an overall precision of 96.09% and overall reliability of 95.19%. Both models had good-excellent sensitivity, with ROC indices of 86.46 and 86.21% for test and learning samples, respectively. The overall lift curve analysis, the ratio between predicted and actual measures and a proxy for model accuracy was significantly high at 99.52%, suggesting that the overall model was valid and accurate.

### Inferential analyses

**Figure 2:**
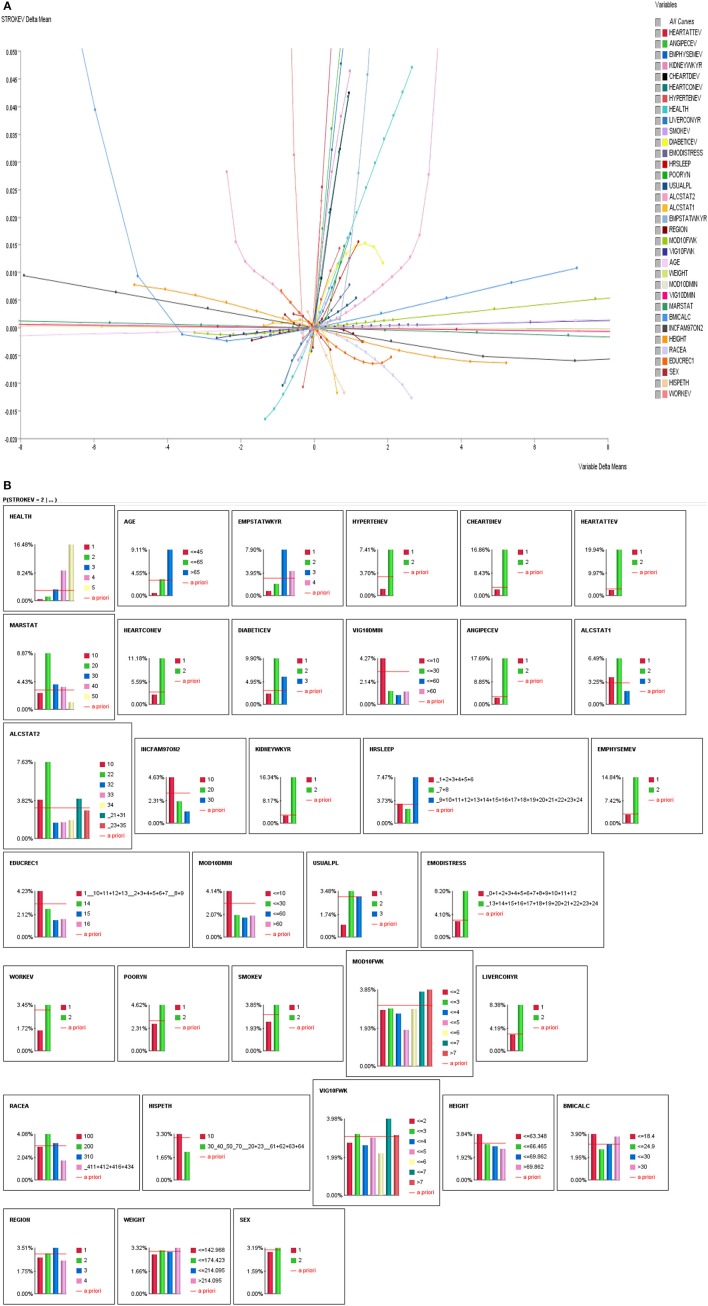
**(A)** Linear and non-linear relationships between history of stroke and stroke correlates. **(B)** Influence Analysis of Variables on self-reported stroke prevalence.

We performed a series of analyses. First, we performed exploratory correlation analyses to illustrate the non-linearity of variables with self-reported stroke making the case that traditional correlation analyses are inadequate to answer the proposed research questions (see Figures 2A,B). Second, we performed observational inferences by applying evidence to different combinations of sleep duration (short, average or long sleep duration), physical activity (intensity: moderate or vigorous, frequency: how many days per week, and duration: how many minutes) to determine the prevalence of self-reported stroke.

In carrying out observational inferences, we fixed the probabilities of all variables, except for stroke, physical activity variables and sleep duration to control for confounding effects. This method assumes that as contingencies are placed on studied variables (physical activity and sleep), the probability distributions of all other variables remain the same. This method is similar to regression-based covariates adjustments, where we isolate the effect of physical activity (PA) and sleep on self-reported stroke prevalence. From this method we are able to see how directly PA and sleep affect the probability distribution of self-reported stroke prevalence. Conversely, we conducted dynamic probability observational inference, where the probabilities of stroke correlates are not fixed as we set contingencies (apply evidence) on studied variables (PA and sleep).

## Results

### Descriptive statistics

The mean age of the sample was 47.79 years ± 18.04, and 48.1% were ≤ 45 years. Of the sample 77.4% were White; 15.9%, Black/African American and the rest “other”; 44.3% male and 55.7% female; and 45.3% reported an annual income less than $35,000 and 24.0% reported an annual family income of $75,000 and up. More than a third of the sample lived in southern states of the U.S. (36.8%). Twenty-nine percent of the sample reported short sleep duration (< 7 h per 24 h period) and 8.7% reported long sleep (≥9 h per 24 h period) duration. Of the entire sample, the overall stroke prevalence rate was 3.07%. We also found that 30.4% of participants who reported a stroke were short sleepers and 20.7% of people who reported a stroke event were long sleepers.

### Exploratory correlations

As indicated above, we performed correlations of all variables with stroke. As seen in Figures 2A,B, majority of the stroke relationships were non-linear, especially the relationships with sleep duration and physical activity. So instead of relying on correlation to determine relationships, Bayesian Belief Network modeling uses mutual information. Table 2 illustrates relationships between self-reported stroke and 34 variables, using mutual information, relative mutual information and Pearson correlation. Of note, the variables that were of most importance to us, physical activity and sleep, either had very small or negative Pearson correlation. We suggest that these smaller or negative relationships occur because traditional correlation metrics are unable of capturing non-linear relationships and relationships of more than two variables, especially those that are associated by way of mediation. Hence, the reason for using BBN modeling is substantiated to better understand non-linear relationships of more than two variables that may be proximal and distal to each other.

**Table 2:** Correlation and Mutual Information of self-reported stroke and 34 correlates.

**Node**	**Mutual information**	**Normalized mutual information**	**Relative mutual information**	**Pearson correlation**	**df**	***p*-value**
HEALTH	0.0238	2.3789%	12.0116%	0.19	4	0.0000%
AGE	0.0219	2.1912%	11.0641%	0.18	2	0.0000%
EMPSTATWKYR	0.0214	2.1365%	10.7878%	0.16	3	0.0000%
HYPERTENEV	0.0183	1.8331%	9.2558%	0.17	1	0.0000%
CHEARTDIEV	0.0127	1.2748%	6.4370%	0.18	1	0.0000%
HEARTATTEV	0.0125	1.2502%	6.3125%	0.19	1	0.0000%
MARSTAT	0.0090	0.9033%	4.5610%	−0.04	4	0.0000%
HEARTCONEV	0.0086	0.8569%	4.3267%	0.14	1	0.0000%
DIABETICEV	0.0083	0.8287%	4.1841%	0.11	2	0.0000%
**VIG10DMIN**	0.0069	0.6854%	3.4607%	−0.07	3	0.0000%
ANGIPECEV	0.0064	0.6388%	3.2254%	0.13	1	0.0000%
ALCSTAT1	0.0061	0.6064%	3.0617%	−0.06	2	0.0000%
ALCSTAT2	0.0060	0.6005%	3.0319%	−0.01	6	0.0000%
INCFAM97ON2	0.0054	0.5423%	2.7383%	−0.08	2	0.0000%
KIDNEYWKYR	0.0045	0.4503%	2.2738%	0.11	1	0.0000%
**HRSLEEP**	0.0038	0.3799%	1.9183%	0.03	2	0.0000%
EMPHYSEMEV	0.0037	0.3665%	1.8504%	0.09	1	0.0000%
EDUCREC1	0.0031	0.3112%	1.5715%	−0.06	3	0.0000%
**MOD10DMIN**	0.0031	0.3111%	1.5709%	−0.04	3	0.0000%
USUALPL	0.0027	0.2666%	1.3460%	0.05	2	0.0000%
EMODISTRESS	0.0016	0.1649%	0.8328%	0.06	1	0.0000%
WORKEV	0.0016	0.1617%	0.8166%	0.04	1	0.0000%
POORYN	0.0011	0.1124%	0.5677%	0.04	1	0.0000%
SMOKEV	0.0011	0.1122%	0.5663%	0.04	1	0.0000%
**MOD10FWK**	0.0009	0.0854%	0.4312%	0.02	6	0.0000%
LIVERCONYR	0.0007	0.0722%	0.3646%	0.04	1	0.0000%
RACEA	0.0007	0.0660%	0.3330%	−0.01	3	0.0000%
HISPETH	0.0006	0.0631%	0.3186%	−0.03	1	0.0000%
**VIG10FWK**	0.0005	0.0488%	0.2464%	0.01	6	0.0000%
HEIGHT	0.0005	0.0482%	0.2432%	−0.02	3	0.0000%
BMICALC	0.0005	0.0463%	0.2337%	0.02	3	0.0000%
REGION	0.0004	0.0354%	0.1788%	−0.01	3	0.0000%
WEIGHT	0.0001	0.0058%	0.0291%	0.01	3	0.0351%
SEX	0.0000	0.0041%	0.0209%	0.01	1	0.0267%

*VIG10DMIN, Duration of Vigorous Physical Activity; HRSLEEP, Total hours sleep; MOD10DMIN, Duration of Moderate Physical Activity (PA); MOD10FWK, Frequency of Moderate PA; VIG10FWK, Frequency of Vigorous PA. Bolded words are variables of main variables in analyses*.

### Differential associations of physical activity and sleep duration on stroke

**Figure 3:**
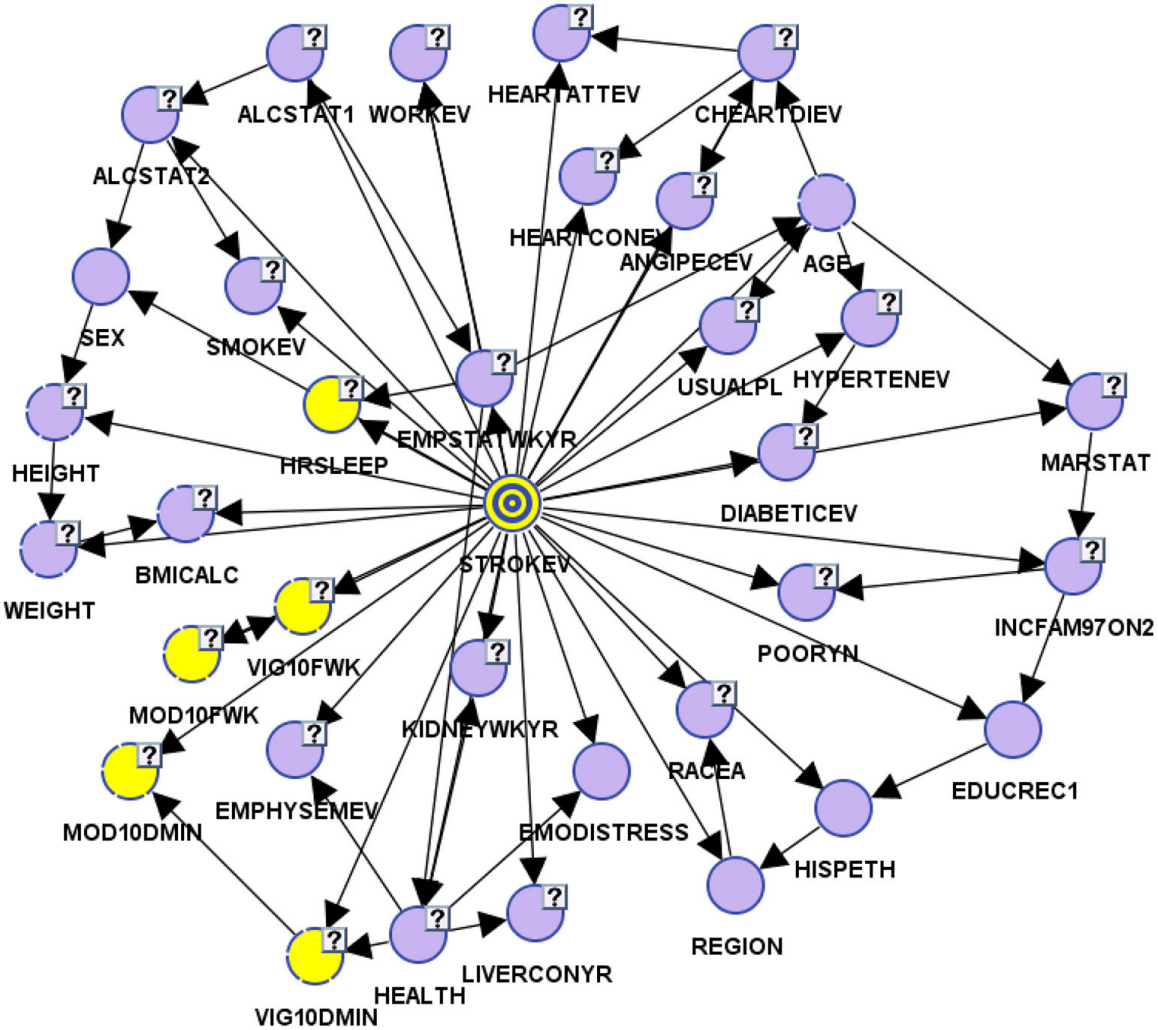
Bayesian belief network model in validation mode to perform observational inference.

**Figure 4:**
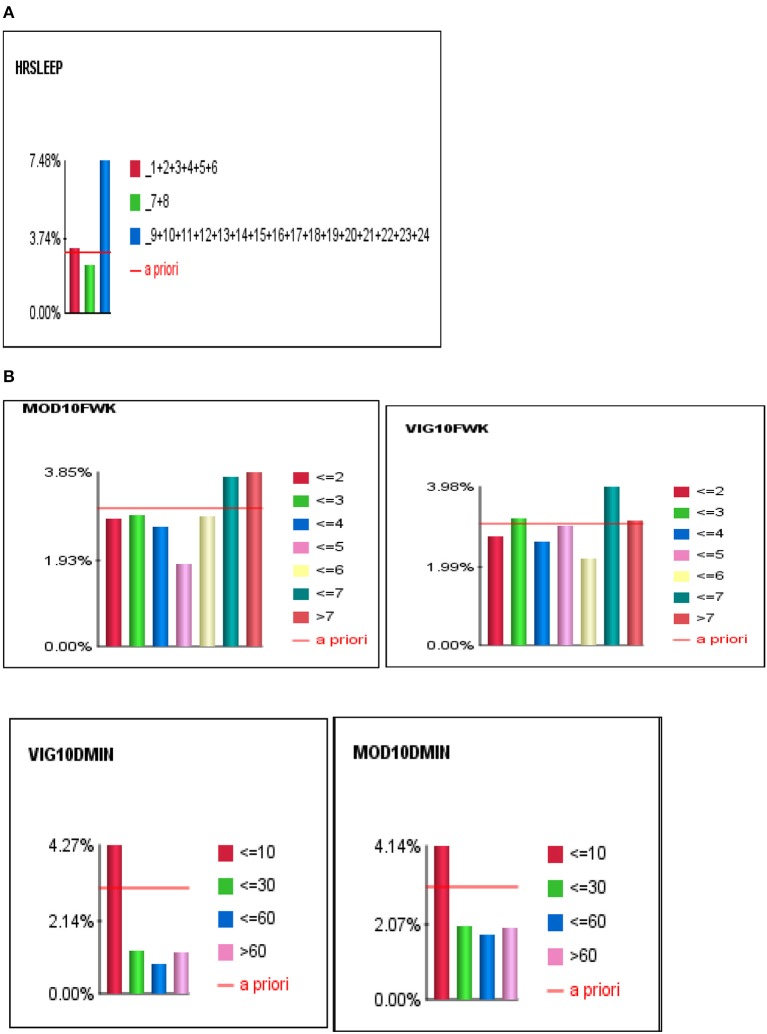
**(A)** The J-shape relationship between Stroke and Sleep Duration. **(B)** U-shape and reverse J-shape relationships between Stroke and Frequency of Moderate and Vigorous Physical Activity.

For Bayesian Belief Network analyses, the prevalence of self-reported stroke among individuals who engaged in < 10 min, 10–30 min, 31–60 min, and >60 min of vigorous physical activity on average was 4.27, 1.26, 0.86, and 1.23%, respectively. While the prevalence of self-reported stroke among individuals who engaged in < 10 min, 10–30 min, 31–60 min, and >60 min of moderate physical activity was 4.14, 1.99, 1.76, and 1.98%, respectively (see Figures 3, 4A,B).

To determine the differential association between sleep duration and self-reported stroke, we applied two conditions to the BBN model, fixed and dynamic probability conditions. Fixing the probabilities of co-variates allowed us to test the independent effect of sleep duration on self-reported stroke. Applying a dynamic condition allowed us to test the joint distribution effects of the machine-learned network and the confounding effects of the other 33 variables on the relationship between sleep duration and stroke. In the fixed model, 3.31% of short sleepers (≤6 hrs.) had a self-reported stroke, 2.57% of average sleepers (7–8 h) had a self-reported stroke, while 4.63% of long sleepers (≥9 h) reported a stroke. In the dynamic model, 3.74% of short sleepers reported a stroke, 2.37% of average sleepers reported a stroke, and 7.48% of long sleepers reported a stroke (146% greater than the national rate of 3.07%) (see Figures 4A,B). Our findings illustrate that long sleep duration compared to short and average sleep durations had a greater association with stroke (*p* < 0.001), regardless of applying fixed or dynamic conditions (see Figure 4A). Our findings also indicate that fixing the probabilities of the other 33 factors may suppress the actual impact sleep duration has on self-reported stroke in the model, as the prevalence of stroke was lower in fixed models vs. dynamic models. Fixed probability conditions provide a more conservative estimate and therefore we utilized this method for all subsequent analyses (see Table 3 and Figure 3).

**Table 3:** Physical Activity and Sleep profiles associated with Stroke across age groups and sex in 2004–2013 National Health Interview Survey.

**Age**	**Sex**	**Physical activity (PA)**	**Frequency of PA**	**Stroke % (PA only)**	**Sleep duration**	**Stroke % (PA and sleep)**
18–45	Male	Moderate activity (31–60 min)	5 times/week	0.19^*^	Short (< 7 h)	0.22
18–45	Female	Moderate activity (31–60 min)	5 times/week	0.20^*^	Short	0.23
18–45	Male	Vigorous activity (31–60 min)	6 times/week	0.12^*^	Short	0.14
18–45	Female	Vigorous activity (31–60 min)	6 times/week	0.13^*^	Short	0.15
18–45	Male	Moderate activity (31–60 min)	5 times/week	0.19	Average (7–8 h)	0.15^***^
18–45	Female	Moderate activity (31–60 min)	5 times/week	0.20	Average	0.16^***^
18–45	Male	Vigorous activity (31–60 min)	6 times/week	0.12	Average	0.10^*^
18–45	Female	Vigorous activity (31–60 min)	6 times/week	0.13	Average	0.10^**^
18–45	Male	Moderate activity (31–60 min)	5 times/week	0.19^***^	Long (> 8hrs)	0.42
18–45	Female	Moderate activity (31–60 min)	5 times/week	0.20^***^	Long	0.45
18–45	Male	Vigorous activity (31–60 min)	6 times/week	0.12^***^	Long	0.26
18–45	Female	Vigorous activity (31–60 min)	6 times/week	0.13^***^	Long	0.27
46–65	Male	Moderate activity (31–60 min)	5 times/week	1.06^**^	Short (< 7hrs)	1.15
46–65	Female	Moderate activity (31–60 min)	5 times/week	1.14^**^	Short	1.23
46–65	Male	Vigorous activity (31–60 min)	6 times/week	0.64^**^	Short	0.71
46–65	Female	Vigorous activity (31–60 min)	6 times/week	0.69^**^	Short	0.76
46–65	Male	Moderate activity (31–60 min)	5 times/week	1.06	Average(7–8hrs)	0.50^***^
46–65	Female	Moderate activity (31–60 min)	5 times/week	1.14	Average	0.88^***^
46–65	Male	Vigorous activity (31–60 min)	6 times/week	0.64	Average	0.50^***^
46–65	Female	Vigorous activity (31–60 min)	6 times/week	0.69	Average	0.53^***^
46–65	Male	Moderate Activity (31–60 min)	5 times/week	1.06^***^	Long (>8 h)	2.60
46–65	Female	Moderate Activity (31–60 min)	5 times/week	1.14^***^	Long	2.79
46–65	Male	Vigorous Activity (31–60 min)	6 times/week	0.64^***^	Long	1.57
46–65	Female	Vigorous Activity (31–60 min)	6 times/week	0.69^***^	Long	1.68
66+	Male	Moderate activity (31–60 min)	5 times/week	3.22^**^	Short (< 7 h)	3.36
66+	Female	Moderate activity (31–60 min)	5 times/week	3.36^**^	Short	3.52
66+	Male	Vigorous activity (31–60 mins)	6 times/week	2.03^*^	Short	2.13
66+	Female	Vigorous activity (31–60 min)	6 times/week	2.11^*^	Short	2.22
66+	Male	Moderate activity (31–60 min)	5 times/week	3.22	Average(7–8 h)	2.60^***^
66+	Female	Moderate activity (31–60 min)	5 times/week	3.36	Average	2.70^***^
66+	Male	Vigorous activity (31–60 min)	6 times/week	2.03	Average	1.63^***^
66+	Female	Vigorous activity (31–60 min)	6 times/week	2.11	Average	1.69^***^
66+	Male	Moderate activity (31–60 min)	5 times/week	3.22^***^	Long (>8 h)	5.67
66+	Female	Moderate activity (31–60 min)	5 times/week	3.36^***^	Long	9.08
66+	Male	Vigorous activity (31–60 min)	6 times/week	2.03^***^	Long	3.62
66+	Female	Vigorous activity (31–60 min)	6 times/week	2.11^***^	Long	3.74

Stroke %, stroke prevalence probability; Stroke % PA only, stroke prevalence probability with physical activity only; Stroke PA and Sleep, stroke prevalence probability with sleep duration and physical activity.

* p < 0.05;

** p < 0.01;

*** *p < 0.001*.

### Differential associations of sleep duration and physical activity on self-reported stroke, age, and sex effects

Participants 18–45 years of age who engaged in 31–60 min of vigorous physical activity six times per week had the lowest self-reported stroke prevalence at 0.12% (males) and 0.13% (females). Of the participants aged 46–65, those who engaged in 31–60 min of vigorous physical activity six times per week had the lowest self-reported stroke prevalence, 0.64% for males and 0.69% for females. Within participants 66 years or older, those who engaged in 31–60 min of vigorous physical activity six times per week had the lowest self-reported stroke prevalence, 2.03% for males and 2.11% for females (see Table 3). Males across all age groups who reported short, average or long sleep durations had a lower self-reported stroke prevalence compared to their female counterparts (see Table 3). We also found that older age groups had a higher prevalence of self-reported strong among males and females (see Table 3).

### Combined associations of sleep duration and moderate and vigorous physical activity on self-reported stroke prevalence, age, and sex effects

Using the observational inference technique, we developed idiosyncratic profiles of physical activity (i.e., duration and frequency of moderate or vigorous exercise per week) and sleep duration (short, average or long sleep duration) that were associated with reduced self-reported stroke prevalence (Table 3). We found that average sleep duration (7–8 h) compared to short or long sleep had a more significant additive and protective effect on reducing self-reported stroke prevalence rates in the entire sample, if participants engaged in either 31–60 min of vigorous physical activity six times per week or if they engaged in 31–60 min of moderate physical activity five times per week (see Table 3).

Of note, men and women aged 18–45 years who reported short sleep duration, 31–60 min. of vigorous physical activity 6 times per week had the lowest self-reported stroke prevalence at 0.10%. While, 46–65 year old men, who reported average sleep duration and engaged in either 31–60 min. of moderate physical activity 5 times per week or 31–60 min. of vigorous physical activity 6 times per week, had the lowest self-reported stroke prevalence of 0.50%. However, of the women 46–65 years old, those who reported average sleep as well as 31–60 min. of vigorous physical activity had the lowest self-reported stroke prevalence of 0.53%. In men and women 66 years or older, those who reported average sleep and 31–60 min of vigorous physical activity 6 times per week had the lowest self-reported stroke prevalence of 1.63% (men) and 1.69% (women) (see Table 3).

## Discussion

### Differential effects of physical activity and sleep duration on stroke

Overall, we found that moderate or vigorous physical activity less than 10 min per week, as well as short or long sleep durations were associated with the highest self-reported stroke prevalence rates which were above the stroke prevalence of the United States (US), which is consistent with previous findings (11). Conversely, moderate or vigorous physical activity greater than 10 min per week and average sleep duration had lower self-reported stroke prevalence rates than the entire sample at 3.07% and for US rates. Specifically, 31–60 min of moderate or vigorous physical activity were associated with the lowest self-reported stroke prevalence rates. We observed a J-shape relationship between sleep duration and self-reported stroke, and a reverse J-shape and U-shape relationships between moderate and vigorous physical activity and stroke (see Figures 4A,B). This means that both short and long sleep durations were associated with self-reported stroke, albeit long sleep being more strongly associated, and engaging in too little (< 10 min) and too much (>60 min) physical activity increased was associated with higher prevalence of ever having a stroke. Lastly, we found that women compared to male counterparts had a higher likelihood of self-reported stroke regardless of physical activity or sleep profiles.

Our finding that there are certain physical activity profiles that are associated with lower self-reported stroke prevalence is supported by literature indicating that physical activity attenuates cardiovascular disease and stroke risk by improving endothelial functioning; increasing high-density lipoprotein (HDL) (otherwise known as “good” cholesterol) and reducing low-density lipoprotein (LDL) (otherwise known as “bad” cholesterol); improving glucose regulation; reducing triglycerides concentration; reducing body mass index; increases plasma tissue plasminogen activator, which helps to breakdown blood clots which are very likely in a stroke; reducing fibrinogen and platelet antiaggregation activity, which in turn increases blood flow; and reducing biological inflammation linked to cardiovascular disease (4–10).

It is clear that the mechanistic relationships between physical activity and self-reported stroke are multifactorial and complex. Physical activity increases the activity of nitric oxide synthase thereby improving endothelial function, reduces left ventricular hypertrophy; stimulates elevations in plasma tissue plasminogen activator and HDL concentrations, and reduces fibrinogen and platelet activity. Aerobic exercise has been shown to enhance glucose regulation and promote reductions in total serum and LDL cholesterol, triglycerides, total body fat, and systemic inflammation (4–10). Additionally, physical activity and exercise helps to prevent obesity, hypertension, dyslipidemia, and the development of type 2 diabetes, all of which increase stroke risk. The U-shaped relationship between self-reported stroke and physical activity may mean that individuals who reported a stroke are less physically active partly due to stroke-related physical impairments and individuals who reported a stroke may engage in more physical activity to minimize risk for secondary stroke occurrence.

Although, short and long sleep durations are associated with self-reported stroke, we believe they do so through different biological pathways. The relationship between short sleep and stroke may be explained by CVD risk markers. For example, a consequence of chronic short sleep is sleep deprivation, which compromises essential homeostatic processes, such as blood pressure dipping, and glucose and triglyceride regulation during sleep (16–21). The resultant of chronic sleep deprivation is generally increased cardiovascular disease risk. Since, short sleep was significantly associated with self-reported stroke, it is likely that stroke may cause some temporary or permanent disruption to sleep duration, architecture, and quality which may result in excessive fatigue and sleepiness (22). Promoting healthy sleep is very important for stroke recovery as it is linked with repair of damaged brain circuitry as a result of a stroke. Based on our findings, a considerable amount of individuals who report short sleep duration are not getting sufficient sleep to recover from stroke as well as are not engaging in adequate physical activity to aid in the brain recovery process (23, 24). From our study we found several combinations of physical activity and healthy/average sleep duration that might protect an individual from secondary stroke occurrence.

However, the relationship between long sleep and stroke has a less clear pathophysiology. There are four possible explanations for the relationship between long sleep duration and stroke. First, long sleep is considered a consequence, as opposed to a cause of stroke (16, 25). Second, the relationship between long sleep and stroke is considered indirect and residual, whereby the negative effects of long sleep on stroke is mediated by the residual effects of sleep disorders, depression, or socioeconomic status (25, 26). Third, pulse wave velocity, a maker of arterial stiffness, significantly moderates the relationship between long sleep and intracerebral hemorrhage (ICH). Additionally, evidence among the elderly at risk for cardiovascular disease indicates that long sleep duration is a determinant of brachial-ankle pulse-wave velocity (27, 28). Lastly, blood pressure variability, a significant predictor of stroke, is also linked to long sleep duration, whereby long sleep duration is associated with increased artery stiffness for individuals with higher visit-to-visit blood pressure variability (28, 29).

### Combinative associations of sleep and physical activity on stroke

Both the American Heart Association and American Stroke Association in 2008 released a joint statement for post-stroke care recommending that stroke survivors should engage in low-to moderate-intensity aerobic activity, muscle strengthening activity, and reduce sedentary behavior. The joint statement emphasized that optimal stroke prevention and post-stroke care should include tailored physical activity and behavioral modification of risk factors. These recommendations were corroborated by one study that showed that the risk of secondary stroke was lowered by 80% with consistent exercise, diet, and use of cholesterol lowering medications, antihypertensive medications, and aspirin (30). The tailored sleep and physical activity profiles in the current study address the need for customized prevention and post-stroke care plans, and highlights that sleep duration and physical activity combined lowered the risk of stroke more than sleep or physical activity separately. Our findings may also mean that adding the two (sleep duration and physical activity) fully optimizes the health benefits of sleep and physical activity, especially among stroke survivors.

Generally, behavioral modification recommendations are generic and are developed for a one-size-fits-all plan. Our findings, based on BBN model, can be used to personalize prevention and post-stroke care behavioral plans to the lifestyles of individuals. For example, an individual who works non-traditional hours due to shift work or multiple jobs will have difficulty adhering to recommended physical activity and sleep guidelines. With the use of our BBN model, an individual can determine how much sleep and/or physical activity and at what frequency and intensity are needed to reduce their stroke risk, primary or secondary prevention. We believe such an approach revolutionizes medicine, making it more precise and personalized– two features that are critical for targeted and effective prevention and treatment of stroke.

### Sex effects

In Table 3, we found that women at all ages who had the same physical activity and sleep profile as their male counterparts had a higher prevalence of self-reported stroke. These findings are consistent with previous research indicating that women had more stroke events than men because of their longer life-expectancy and higher stroke incidence at older ages (31). Additionally, women compared to men receive less post-stroke medical care which compromises recovery and rehabilitation and leads to poorer functional outcomes, quality of life and prognosis (31). To mitigate these sex effects, tailored sex-specific post-stroke treatments are encouraged.

### Limitations

There are a few limitations that bear mentioning. Since the NHIS dataset does not have information on specific stroke type and date of stroke, we were unable to assess the relationships between type of stroke, stroke date, sleep duration, and physical activity. Even though we included hypertension as a risk factor in the model, the lack of other clinical stroke risk factors, such as atrial fibrillation and atherosclerosis (known stroke risk factors), limited our ability to investigate how sleep and physical activity dynamically influenced these clinical risk factors. Also, we were not able to investigate the differential association certain types of physical activities have on stroke. Additionally, the cross-sectional design of the study highlights our inability to make causal claims between sleep duration, physical activity, and stroke. Future studies should investigate the longitudinal effect sleep duration and physical activity have on stroke, and whether sleep and physical activity reduce stroke risk. Lastly, the use of Bayesian Belief Network modeling relies heavily on the given data, as the model is generated based on the evidence at hand. Since there were only 3.07% stroke cases, which is consistent with national rates, applying multiple conditions to the model at the same time reduces the sample size and power. To safeguard against this possibility, we fixed the probabilities of all the factors except the target variables, as well as conducted several validation analyses to ensure that all inferences presented had adequate inferential power.

## Conclusion

To our knowledge, our study is one of the first to investigate the omnidirectional relationships of sleep duration (short sleep, average sleep, and long sleep durations), physical activity (intensity, duration, and frequency), and self-reported stroke, using machine learning modeling techniques. Through this innovative approach, we found that long sleep duration compared to short sleep duration was more associated with self-reported stroke prevalence in the U.S. Although previous studies found a similar association, it was believed that comorbid medical conditions might be driving the association between physical activity, long sleep, and stroke. Since our inclusion of likely medical comorbidities in the Bayesian Belief Network model accounts for these associations, we are confident that the association we found was independent of medical comorbidities. We also found that several sleep duration and physical activity combinations were associated with reduced stroke. If you are a short sleeper (< 7 h), average sleeper (7–8 h), or long sleeper (>8 h), engaging in 60 min of moderate physical activity (leisure walks, cycling, and light garden work) 5 times for the week had the least stroke risk. However, engaging in 31–60 min of vigorous physical activity (leisure walks, cycling, and light garden work) 6 times for the week was associated with a lower self-reported stroke prevalence than moderate physical activity. These findings indicate that if an individual is unable to get average sleep duration (which is associated with lowest self-reported stroke prevalence compared to short or long sleep duration), engaging in moderate physical activity 31–60 min may protect them from a stroke. Future studies are needed to establish the shared and complementary biological pathways by which physical activity and sleep reduce stroke risk.

## Author contributions

AS developed the idea of the paper. AS and DH led the paper and played key roles in the development of all components of the manuscript. SW and AR developed the introduction section, DH and AS performed the analyses. AS, DH, SW, RJ, AR, FZ, and GJ-L developed and edited the Discussion section. All authors have read the manuscript and approve of the final product.

### Conflict of interest statement

The authors declare that the research was conducted in the absence of any commercial or financial relationships that could be construed as a potential conflict of interest.
